# Maternity and Mothering

**Published:** 1911-11-04

**Authors:** 


### MATERNITY AND MOTHERING.

(Inquiries and Correspondence are invited)

### The Care of Children's Teeth.—III.

Having dealt with the proper diet of nurslings and young children, the next point for consideration is the care of the temporary teeth from the time at which they are fully erupted onwards; roughly, that is, from the age of two years.

Diet is still of paramount importance; though, as will be seen presently, there are other matters that require attention also. Here, again, no one will go far wrong who follows the advice of Drs. Wheatley and Wallace, as given at the Birmingham meeting of the British Medical Association. Children should have three meals a day; and nothing between meals, except pure water if they complain of thirst. Each meal should contain a sufficiency of food which by its nature requires full mastication and cannot be bolted. This last habit, if detected, is to be firmly discouraged. Toast, baked bread, or crusty rolls are excellent for ensuring sufficient mastication; and crust is always preferable to crumb in all kinds of bread.

Then the arrangement of each meal is important. No meal ought to conclude with carbohydrate foodstuffs of such a nature that they are likely to lodge in or stick to the teeth. Therefore no meal should end with jam or marmalade, nor with sweet puddings or any food containing large quantities of sugar. The habit of sprinkling powdered sugar on children's bread is also to be deprecated very strongly. If such foods are given, they should be followed by fresh fruit of some kind; and of all fruits which cleanse the mouth and teeth apples are the best.

### The Abuse of Sweetmeats.

It has been remarked that the last few years have seen an enormous increase in the number of shops devoted to the sale of sweetmeats; and this is probably true. Yet it is also probably true that in consequence the sweetmeats of to-day are cleaner and less likely to contain directly injurious ingredients than those of twenty years ago.

At the same time, there is no doubt that both adults and children consume a great many more sweets nowadays than used to be the case; and that this habit is one which is positively conducive to dental caries. Mr. Hopewell-Smith goes so far as to advise that sweetmeats should be rigidly denied to the young by parents, and takes a very serious view of the mischief caused by lollipops. In this crusade it is obvious that the parents have first to be converted; and even then the modern parent is so little accustomed to exact proper discipline from his (or her) children that there is a risk of his prohibitions being neglected, or of the sweets being consumed in secret instead of openly. Still parents have after all control over finance, and can easily, if they will, take steps to secure compliance with their wishes.

Fortunately there is evidence that the craving of the modern child for sweets is partly an acquired taste and partly the result of incorrect feeding. A child brought up on the dietary suggested in this and the preceding article will be found not to develop this craving to any great extent. Children who are correctly dieted may be allowed to buy and eat sweets, so long as none are eaten between meals. It will be found that their appetite for sweets will in most cases be small, and that there will be no need for disciplinary measures to enforce strict moderation.

### Dental Supervision.

Lastly, but by no means least in importance, comes the need for careful and constant supervision. All irregularities in eruption and arrangement of the teeth must be corrected; for they are fertile causes of impaction or stagnation of food particles, and hence of fermentation and decay. (Incidentally it is worth noting that breast-fed children display dental abnormalities much less frequently than the bottle fed.) Mouth breathing is another disposing cause of caries, and hence adenoids must be removed, or, better still, prevented.

Any teeth which have already decayed must be properly treated by a competent dentist; not only to preserve them as far as possible, but also with the object of protecting the sound teeth from a similar fate.

On the subject of the tooth-brush a great deal has been written of late. Some, as Drs. Smale and Carmalt-Jones, condemn it as a septic and useless instrument; and demand that it shall be either boiled or soaked in very strong antiseptics every time after usage. Others still hold that regularity in the use of the tooth-brush is one of the cardinal virtues, and will be rewarded by an undecayed set of teeth. Probably the truth lies somewhere between the two extremes; the tooth-brush regularly used is valuable, but not so essential as a correct dietary. Antiseptic tooth-powders and mouth washes are of little value, if not actually deleterious; plain precipitated chalk is useful for keeping the teeth of smokers white, and others may use it at discretion. The main lesson is to have only soft brushes and to use them gently. Quill tooth-picks are useful sometimes to clear food debris out of interstices.

The methods here outlined are not based on theoretical considerations alone. They have been tested and proved to be effective, and are not merely the latest fad in dental surgery. An editorial lately published in *School Hygiene* takes up a line of mild protest against the introduction of these new methods, and comments on the diverging views of those who decry and those who applaud the use of the tooth-brush by school children. While admitting the justice of some of the remarks made, it must be urged that, while children properly dieted may not need tooth-brushes to ward off caries, yet few children are thus fortunately situated. So that the inconsistency of the dentists may be in some ways more apparent than real.

